# Regulation of mTORC1 Signaling by pH

**DOI:** 10.1371/journal.pone.0021549

**Published:** 2011-06-29

**Authors:** Aruna D. Balgi, Graham H. Diering, Elizabeth Donohue, Karen K. Y. Lam, Bruno D. Fonseca, Carla Zimmerman, Masayuki Numata, Michel Roberge

**Affiliations:** 1 Department of Biochemistry and Molecular Biology, University of British Columbia, Vancouver, British Columbia, Canada; 2 The Centre for Drug Research and Development, Vancouver, British Columbia, Canada; Texas A&M University, United States of America

## Abstract

**Background:**

Acidification of the cytoplasm and the extracellular environment is associated with many physiological and pathological conditions, such as intense exercise, hypoxia and tumourigenesis. Acidification affects important cellular functions including protein synthesis, growth, and proliferation. Many of these vital functions are controlled by mTORC1, a master regulator protein kinase that is activated by various growth-stimulating signals and inactivated by starvation conditions. Whether mTORC1 can also respond to changes in extracellular or cytoplasmic pH and play a role in limiting anabolic processes in acidic conditions is not known.

**Methodology/Findings:**

We examined the effects of acidifying the extracellular medium from pH 7.4 to 6.4 on human breast carcinoma MCF-7 cells and immortalized mouse embryo fibroblasts. Decreasing the extracellular pH caused intracellular acidification and rapid, graded and reversible inhibition of mTORC1, assessed by measuring the phosphorylation of the mTORC1 substrate S6K. Fibroblasts deleted of the tuberous sclerosis complex TSC2 gene, a major negative regulator of mTORC1, were unable to inhibit mTORC1 in acidic extracellular conditions, showing that the TSC1–TSC2 complex is required for this response. Examination of the major upstream pathways converging on the TSC1–TSC2 complex showed that Akt signaling was unaffected by pH but that the Raf/MEK/ERK pathway was inhibited. Inhibition of MEK with drugs caused only modest mTORC1 inhibition, implying that other unidentified pathways also play major roles.

**Conclusions:**

This study reveals a novel role for the TSC1/TSC2 complex and mTORC1 in sensing variations in ambient pH. As a common feature of low tissue perfusion, low glucose availability and high energy expenditure, acidic pH may serve as a signal for mTORC1 to downregulate energy-consuming anabolic processes such as protein synthesis as an adaptive response to metabolically stressful conditions.

## Introduction

Acidification of the extracellular space or the cytoplasm is observed in a number of physiological and pathological conditions associated with intense energy expenditure, metabolic disturbance or hypoperfusion. During high intensity exercise, the intracellular pH of the skeletal muscle can transiently decrease from a normal of 7.0 to as low as 6.2 due to high metabolic acid production [1]. Elevated levels of acetoacetate and D-β-hydroxybutyrate during starvation- or diabetes-induced ketogenesis can decrease the blood pH from a normal of 7.4 to 7.0–7.1 [2]. Hypoxia and ischemia in animal models induce major intracellular and extracellular acidification, with decreases reaching 0.8–1.2 pH units in the brain [3]. Cardiac arrest also triggers intracellular acidification that rapidly reaches pH values of 6.0–6.5 in the myocardium and the cerebral cortex and returns to normal values within a few minutes upon resuscitation [4], [5]. Low extracellular pH is a hallmark of solid tumours with values as low as 6.2 [6], [7], [8] probably resulting from poor vascularization and the heavy reliance of tumour cells on glycolysis for ATP production [9], [10].

Important cellular processes such as protein synthesis, growth and proliferation are negatively impacted by even mild acidification [11], [12], [13], [14], [15], [16]. It is unclear whether this reflects an adaptive cellular response to adverse conditions or merely an inability of cells to function normally at suboptimal pH. The Ser/Thr kinase mTOR is a master regulator of cell metabolism, growth, survival and proliferation [17] that is active when conditions are conducive for growth. mTOR is found in two functionally and structurally distinct multiprotein complexes, mTORC1 and mTORC2, which signal via distinct effector pathways [18]. mTORC1 is negatively regulated by the TSC1–TSC2 complex which integrates several major upstream mTORC1 regulatory signals including growth factors, low energy (ATP) and oxygen depletion [19], [20]. This study examines the role of the mTORC1 signaling pathway in the cellular response to changes in pH. The results show that mTORC1 activity is rapidly and reversibly inhibited by acidification through a TSC complex-dependent mechanism and identify pH as a further environmental input into mTORC1 signaling.

## Results

### Reversible inhibition of mTORC1 signaling by extracellular acidic pH

**Figure 1:**
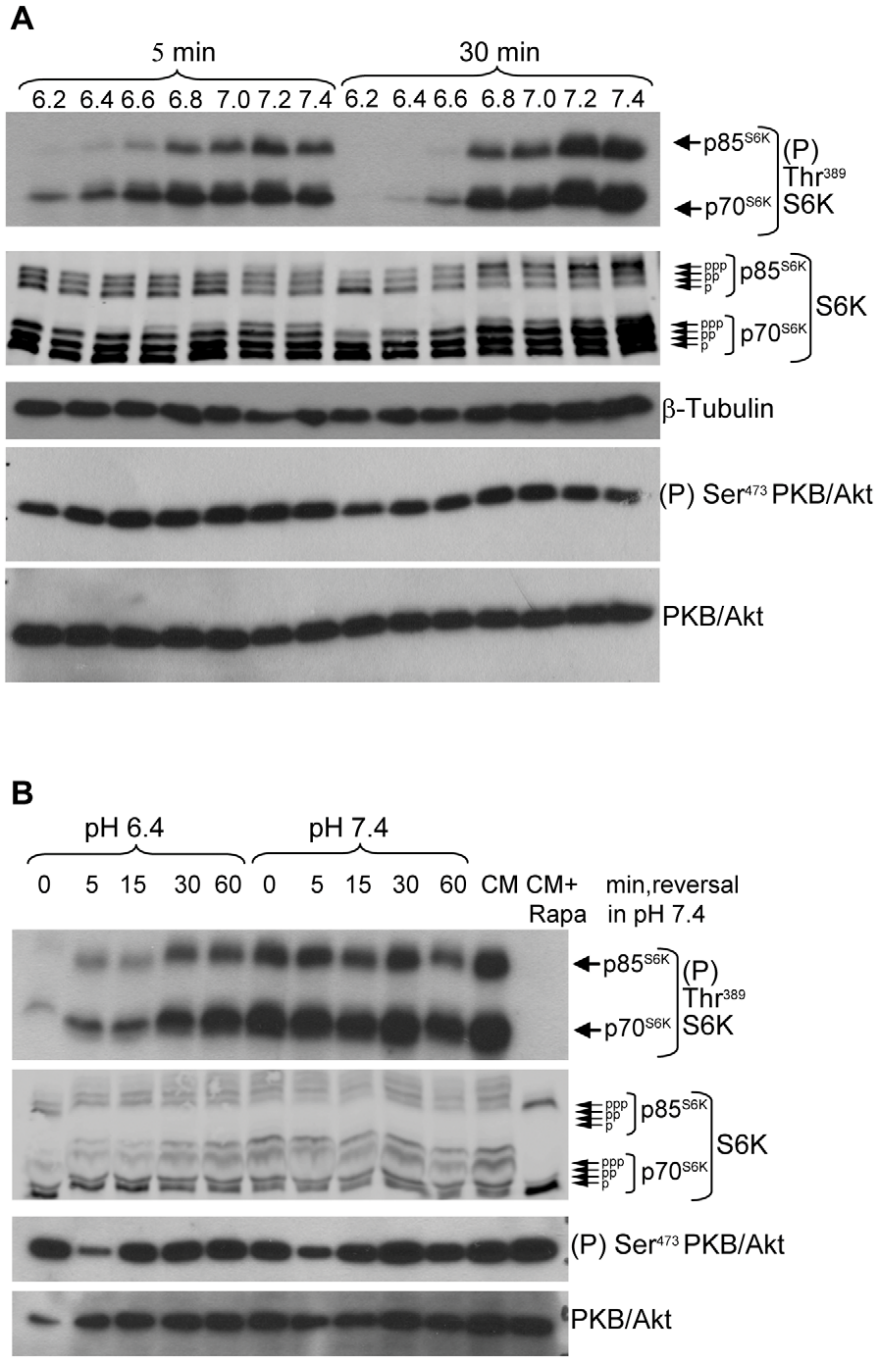
Rapid and reversible inhibition of mTORC1 signaling by acidic extracellular pH. A, MCF-7 cells were exposed for 5 min or 30 min to cell culture medium buffered to the indicated pH values. Lysates were analysed for mTORC1 activation by western blotting using antibodies to phospho-Thr^389^ S6K and to S6K and for mTORC2 activation using antibodies to phospho-Ser^473^ PKB/Akt and to PKB/Akt. Tubulin immunodetection was used as a protein loading control. B, MCF-7 cells grown in normal cell culture medium were exposed to medium buffered to pH 6.4 or 7.4 for 30 min. The medium was removed and replaced with medium buffered to pH 7.4 and samples were harvested immediately (0 min) or at the indicated times. In parallel, cells were exposed or not to the mTORC1 inhibitor rapamycin (Rapa, 30 nM) for 30 min in normal cell culture medium (CM). Lysates were analysed by immunoblotting. Note that changing the cell culture medium caused a minor and transient decrease in PKB/Akt Ser^473^ phosphorylation that was independent of acidic pH. Results shown in all figures are representative of three or more independent experiments.

The extracellular pH of solid tumours is commonly acidic, ranging from 6.2–7.0 [8] and blood pH can drop to below 7.0 during severe metabolic acidosis [2], [21]. To examine whether acidic extracellular pH in this range affects mTORC1 signaling, human breast carcinoma MCF-7 cells were incubated for 5 min or 30 min in cell culture medium buffered to different pH values. Phosphorylation of the mTORC1 substrates p70 S6K and p85 S6K was monitored with a phosphospecific antibody and by examining the electrophoretic mobility of S6K, which is reduced by phosphorylation. Exposure of cells to pH 6.2–6.6 elicited a rapid and pronounced decrease in mTORC1 signaling that was detectable within 5 min and essentially complete by 30 min (Fig. 1A). Exposure to medium buffered to pH 6.8–7.0 caused partial mTORC1 inhibition at 30 min while high mTORC1 activity was observed at physiological pH values of 7.2 and 7.4 (Fig. 1A).

If mTORC1 is a component of an adaptive response to pH change, its inhibition by acidic pH should be reversible and its activity should return to normal upon reestablishment of physiological pH conditions. Cells were incubated in medium buffered to pH 6.4 or 7.4 for 30 min, after which time the medium was replaced with medium buffered to pH 7.4 and mTORC1 activity was examined at different times. After 30 min exposure to pH 6.4, mTORC1 activity was strongly repressed while it remained high at pH 7.4 (Fig. 1B, lanes 0). When cells were transferred from pH 6.4 to pH 7.4, mTORC1 activity increased detectably within 5 min and returned to normal values within 30 min (Fig. 1B). In the control experiment, transfer of cells from pH 7.4 to pH 7.4 caused no change in mTORC1 activity, as expected (Fig. 1B).

mTOR is also the catalytic subunit of mTORC2 that phosphorylates PKB/Akt at Ser^473^ [22], [23], [24]. Examination of PKB/Akt Ser^473^ phosphorylation in the same samples revealed that exposure to acidic pH did not inhibit mTORC2 (Fig. 1A), indicating that acidic extracellular pH does not directly inhibit the catalytic activity of mTOR but probably impacts an upstream mTORC1 regulatory signal. Therefore, acidic extracellular pH values commonly found in solid tumours or during acidosis can rapidly and reversibly inhibit mTORC1 signaling.

### Effect of extracellular acidification on intracellular pH

**Figure 2:**
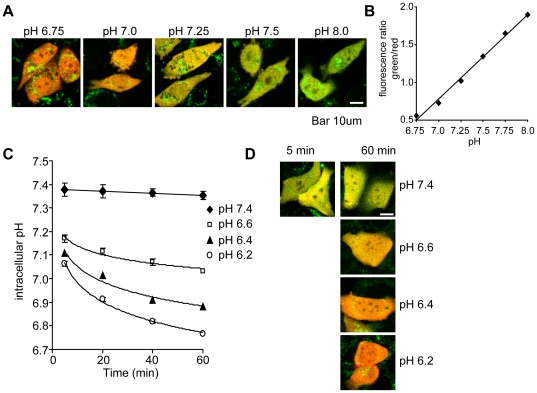
Changes in intracellular pH in cells exposed to extracellular acidification. A, Confocal images of CHO cells transfected with mCherry/de4GFP under pH clamp (high [K**^+^**]/Nigericin) from pH 6.75 to pH 8.0. An increase in green fluorescence indicates a pH shift in the alkaline direction. Scale bar = 10 µm. B, The relationship between mCherry/de4GFP fluorescence ratio and pH was determined. C, Intracellular pH in mCherry/de4GFP transfected MCF-7 cells was monitored with time after the addition of cell culture medium buffered to the indicated pH values. N>70 cells per condition. D, Sample images of MCF-7 cells exposed to control medium for 5 or 60 min or acidified media for 60 min are shown. A red shift resulting from a loss of green fluorescence indicates intracellular acidification. Scale bar = 10 µm.

Cells might respond by directly sensing extracellular pH and/or via intracellular sensors if intracellular pH is affected by extracellular acidification. We next monitored the effects of varying extracellular pH on intracellular pH in cells transfected with a novel fluorescent ratiometric reporter consisting of pH insensitive mCherry fused to the de4GFP pH-sensitive variant of GFP [25]. Calibration under pH-clamp conditions using the protonophore nigericin (high K^+^, 10 µM nigericin) showed that intracellular acidification causes a red shift in fluorescence (Fig. 2A) that is linear between pH 6.75 and 8.0 (Fig. 2B). MCF-7 cells expressing mCherry/de4GFP were exposed to medium buffered to pH 6.2, 6.4, 6.6 or 7.4 and changes in intracellular pH were monitored over time. At normal external pH of 7.4, the intracellular pH was slightly lower (7.35–7.40) for the 1 h observation period (Fig. 2C). Extracellular acidification caused a time-dependent decrease in intracellular pH. Within 5 min exposure to extracellular pH of 6.2, 6.4 or 6.6, the internal pH decreased to 7.06±0.02, 7.11±0.02 or 7.17±0.02, respectively. After 1 h, the internal pH had further dropped to 6.77±0.01, 6.88±0.01 and 7.03±0.01 respectively (Fig. 2 C). Composite images of the MCF-7 cells show the decrease in intracellular green/red fluorescence ratio with decreased extracellular pH (Fig. 2D). The observation that extracellular acidification causes rapid changes in intracellular pH raises the possibility that signals controlling mTORC1 might originate from inside the cell, while not excluding an involvement of cell surface sensors.

### mTORC1 inactivation by acidification requires TSC2

**Figure 3:**
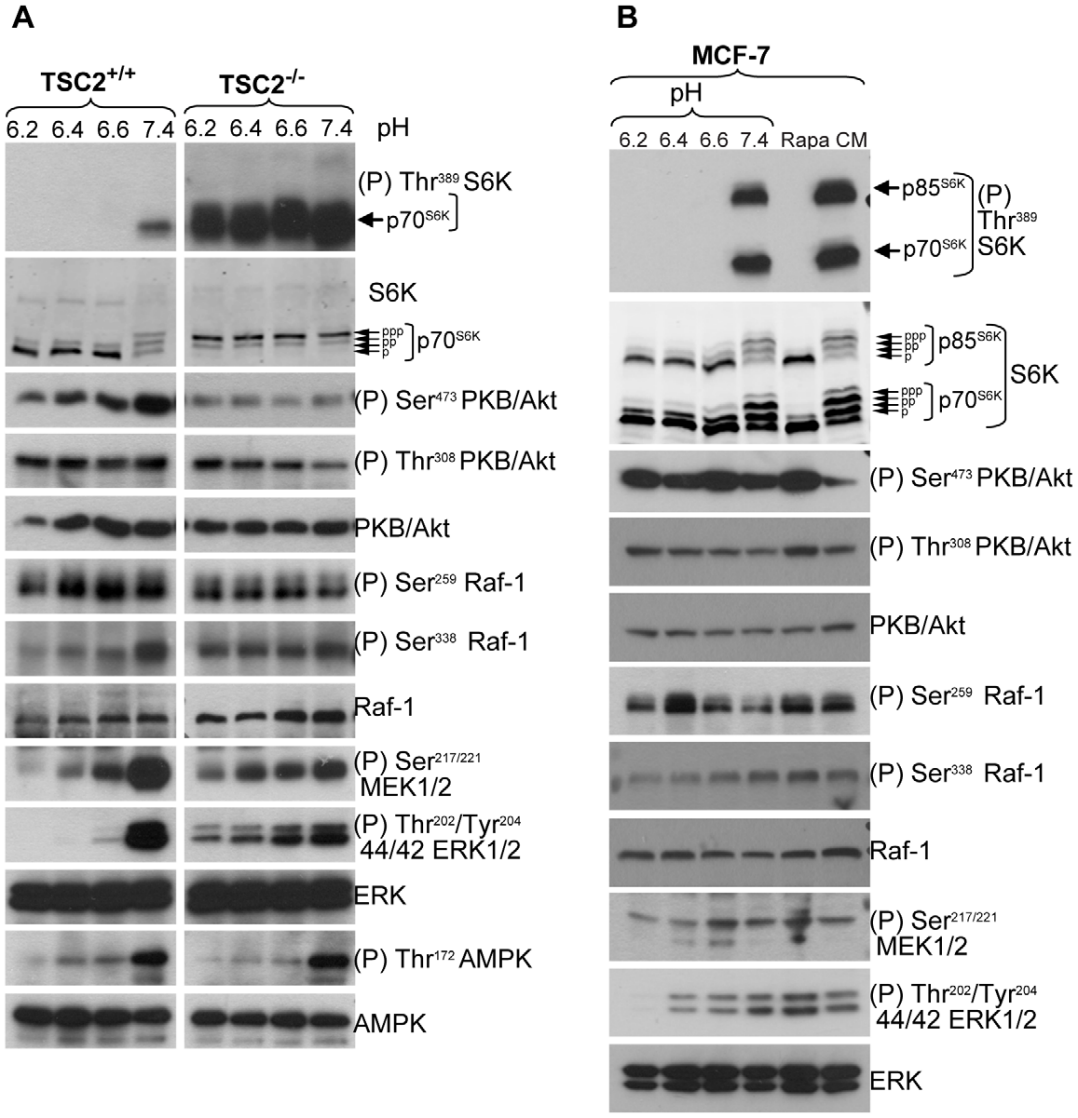
Effect of extracellular acidification on mTORC1 control pathways. TSC2^+/+^ and TSC2^−/−^ MEFs (A) or MCF-7 cells (B) were exposed for 1 h to medium buffered to the indicated pH values or to 30 nM rapamycin for 1 h and lysates were analysed by western blotting using the indicated antibodies.

The TSC1–TSC2 complex plays a central role in control of mTORC1 by integrating signaling from several upstream pathways to negatively regulate mTORC1 [20]. Immortalized mouse embryo fibroblasts (MEF) deleted of both TSC2 alleles show high mTORC1 activity compared to their wild-type counterparts (Fig. 3A), consistent with TSC2's role as a negative regulator of mTORC1 [20]. To determine whether a functional TSC1–TSC2 pathway is required for negative regulation of mTORC1 by acidic pH, TSC2^+/+^ and TSC2^−/−^ MEFs were exposed for 1 h to medium at different pH values. Acidic extracellular pH (6.2–6.6) completely inhibited mTORC1 in TSC2^+/+^ MEFs (Fig. 3A). By contrast, when TSC2^−/−^ MEFs were exposed to acidic pH, no decrease in mTORC1 activity was detected, as seen from a lack of reduction in the phospho S6K Thr^389^ signal and a lack of change in the electrophoretic mobility of S6K (Fig. 3A).

By comparison, the phosphorylation of PKB/Akt at its two activating sites Ser^473^ and Thr^308^ by mTORC2 and PDK1, respectively, was not strongly affected by acidic pH in TSC2^+/+^ or TSC2^−/−^ cells (Fig. 3A). These results show that the TSC1–TSC2 complex is required for cells to inhibit mTORC1 when exposed to acidic pH and may implicate it in relaying upstream pH-sensing signals to mTORC1.

### Inhibition of the Raf-MEK-ERK pathway by extracellular acidic pH

The TSC1–TSC2 complex acts as a hub that integrates different upstream signals to control mTORC1 activity [20]. Growth factor signaling to the TSC1–TSC2 complex takes place in part via activation of the Ras-Raf-MEK-ERK signaling cascade. Both ERK and the downstream kinase p90RSK can phosphorylate TSC2 and inhibit the TSC1–TSC2 complex, leading to activation of mTORC1 [26]. Exposure of TSC2^+/+^ cells to acidic pH caused a profound inhibition of the phosphorylation of ERK at its activation sites Thr^202^/Tyr^204^ (Fig. 3A). Cells lacking TSC2 showed a lower basal level of ERK phosphorylation at physiological pH than cells expressing TSC2, possibly as a consequence of aberrantly high mTORC1 activity [27], and also a more modest reduction in ERK phosphorylation at acidic pH. ERK is phosphorylated at Thr^202^/Tyr^204^ by MEK, indicating that acidic extracellular pH inactivates MEK. MEK is itself activated by phosphorylation at Ser ^217/221^ by Raf kinases. Exposure to acidic extracellular pH also caused a strong reduction in MEK Ser ^217/221^ phosphorylation in TSC2^+/+^ cells (Fig. 3A). TSC2^−/−^ cells showed a lower basal level of MEK phosphorylation at physiological pH and a much smaller reduction in MEK phosphorylation at acidic pH (Fig. 3A). Human breast carcinoma MCF-7 cells exposed similarly to acidic medium showed essentially complete inhibition of mTORC1 and decreased phosphorylation of ERK1/2 at Thr^202^/Tyr^204^, MEK1/2 at Ser^217/221^, especially at pH 6.2 (Fig. 3B), whereas phosphorylation of the mTORC2 substrate AKT was not affected (Fig. 3B).

MEK is phosphorylated and activated by Raf. The mechanisms by which Raf is activated in response to growth factors are still incompletely understood but involve phosphorylation at Ser^338^ of Raf-1, possibly by autophosphorylation [28]. Raf-1 Ser^338^ phosphorylation was significantly reduced at acidic pH in TSC2^+/+^ cells (Fig. 3A) and MCF-7 cells (Fig. 3B). Similar to ERK and MEK phosphorylation, Raf-1 Ser^338^ phosphorylation was lower in cells lacking TSC2 and its activity was not reduced by acidic extracellular pH. Raf is inactive when cytosolic and recruitment to the plasma membrane is required for activation [29], [30]. Subcellular fractionation experiments and immunofluorescence microscopy indicated that acidic extracellular pH did not prevent membrane association of Raf-1 (not shown).

**Figure 4:**
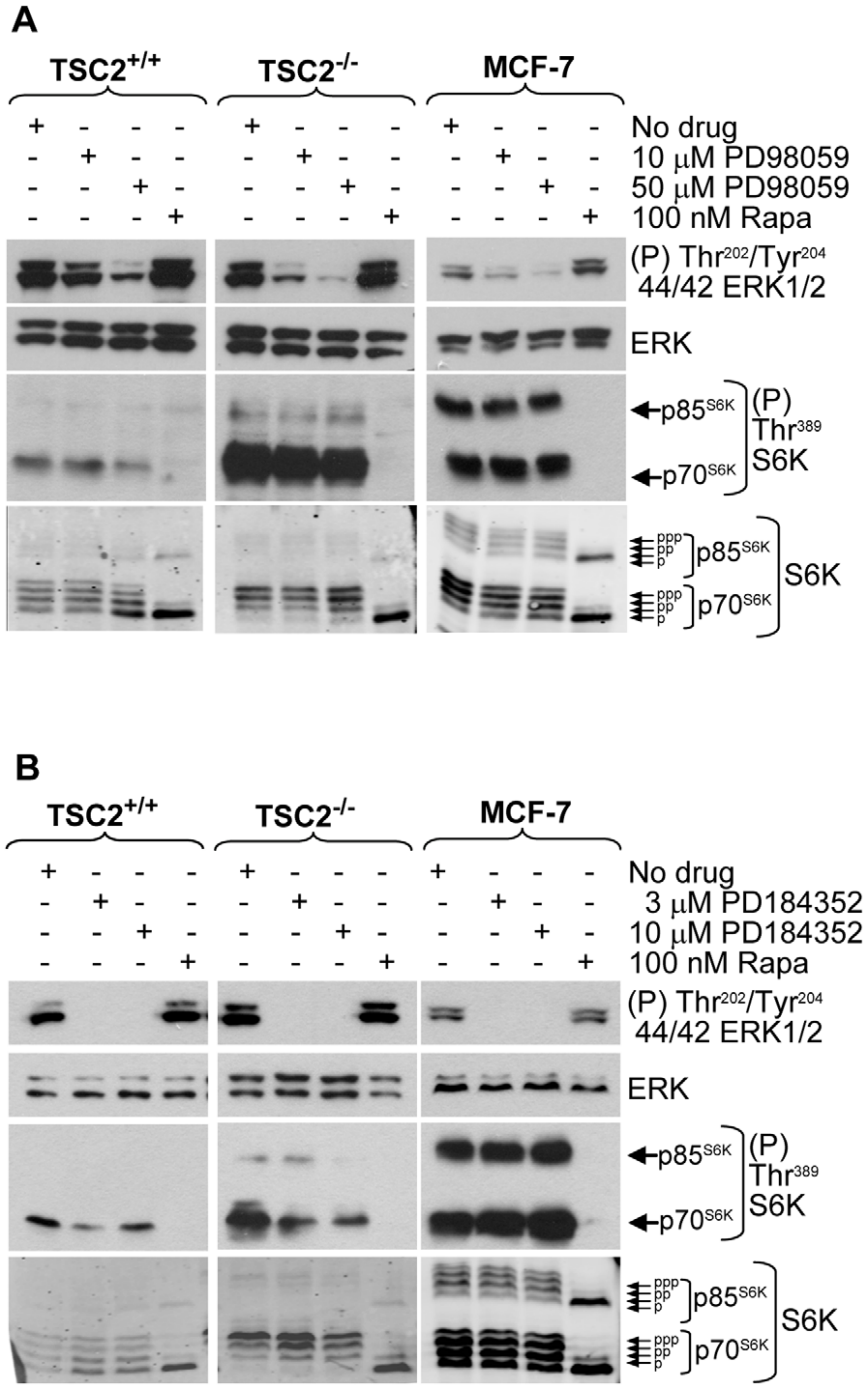
Effect of MEK inhibitors on mTORC1 signaling. Cells were exposed to the MEK inhibitors PD98059 or PD184352 or to rapamycin for 1 h in normal cell culture medium and lysates were analysed by western blotting using the indicated antibodies.

To determine the extent to which MEK-ERK inactivation contributes to mTORC1 inhibition, MEK was inhibited using drugs. In all three cell lines, PD98059 (50 µM) strongly inhibited ERK-activating Thr^202^/Tyr^204^ phosphorylations (Fig. 4A) and PD184352 (3 and 10 µM) caused complete inhibition (Fig. 4B). Both inhibitors caused a modest reduction in mTORC1 activity in TSC2^+/+^ cells, seen by decreased phospho-S6K antibody reactivity and increased S6K electrophoretic mobility (Fig. 4). In TSC2^−/−^ and MCF-7 cells, PD98059 and PD184352 caused little or no decrease in mTORC1 signaling despite efficiently inhibiting ERK phosphorylation (Fig. 4). Together, these data indicate that in TSC2^+/+^ cells that display normal levels of mTORC1 signaling, acidic extracellular pH effectively inactivates the Raf-MEK-ERK pathway but inhibition of the Raf-MEK-ERK pathway contributes only modestly to inhibition of mTORC1 signaling. The results also indicate that in TSC2^−/−^ MEFs and MCF-7 cells displaying abnormal mTORC1 regulation, inactivation of MEK-ERK does not significantly contribute to mTORC1 inhibition by acidic pH.

### Effect of acidic extracellular pH on other mTORC1control pathways

Growth factors also signal to the TSC1–TSC2 complex via the PI3K/Akt pathway.

Binding of growth factors to cell surface receptors results in recruitment of PI3K to the membrane. The resulting generation of PtdIns(3,4,5)P3 recruits Akt to the plasma membrane where it is activated via phosphorylation at Thr^308^ by PDK1 and at Ser^473^ by mTORC2. Akt then directly phosphorylates TSC2 to inactivate the TSC1–TSC2 complex [20]. Exposure to acidic pH did not affect Akt phosphorylation at either Thr^308^ or Ser^473^ in TSC2^−/−^ and TSC2^+/+^ MEFs (Fig. 3A) or MCF-7 cells (Fig. 1A, 3B), indicating that the PI3K-Akt pathway does not participate in pH sensing.

AMPK is a low-energy sensor; in response to ATP depletion it is activated by phosphorylation at Thr^172^ and inhibits mTOR-dependent signaling by phosphorylating TSC2 [31], [32], [33]. Phosphorylation at Thr^172^ was not stimulated by acidic extracellular pH. AMPK Thr^172^ phosphorylation actually decreased at acidic pH in both TSC2^+/+^ and TSC2^−/−^ cells (Fig. 3A). Transcriptional induction of REDD1, regulated by the HIF-1α transcription factor, activates TSC2 to negatively regulate mTORC1 in response to hypoxia [34], [35]. This response requires *de novo* transcription of the Redd1 gene [35]. Since we observed effects of acidic extracellular pH on mTORC1 signaling within 5 minutes, it is highly unlikely that pH affects TSC1–TSC2 through increased transcription of REDD1, a response that takes over 3 hours [35], [36].

**Figure 5:**
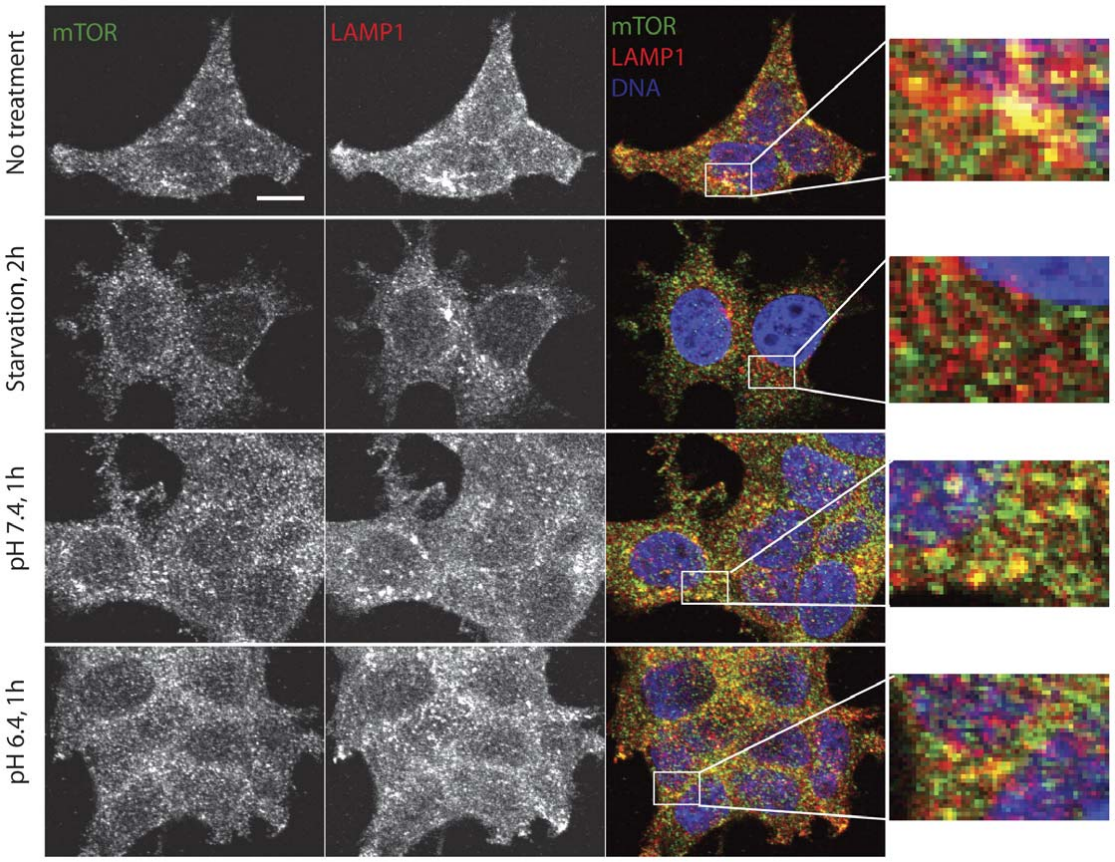
Effect of extracellular acidification on the cytoplasmic localization of mTOR and lysosomes. MCF-7 cells were left untreated, exposed to medium buffered to pH 6.4 or 7.4 for 1 h, or exposed to HBSS for 2 h and immunostained using antibodies as shown. Yellow signal indicates co-localization of mTOR and LAMP-1. Bar, 10 µm.

Starvation, which inhibits mTORC1, has recently been shown to alter the localization of lysosomes within the cytoplasm and the association of mTOR with lysosomes [37], [38], [39]. To determine whether extracellular acidification caused similar changes, cells were exposed to pH 6.4 or 7.4 for 1 h and mTOR and the lysosomal marker LAMP1were visualized by immunofluorescence microscopy. Exposure to pH 6.4 did not affect the cytoplasmic distribution of lysosomes or the extent of co-localization of mTOR with LAMP-1, which was similar in cells maintained in complete culture medium or exposed to medium buffered to pH 7.4 or 6.4 (Fig. 5). By contrast, when cells were starved of amino acids and serum, extensive delocalization of mTOR from LAMP-1 took place (Fig. 5), as previously reported [38], [39].

### Effect of acidic extracellular pH on mTORC1 activation by serum

The experiments described thus far were carried out in the presence of serum, which contains growth factors that maintain high mTORC1 activity, and show that exposure to acidic extracellular pH can rapidly interrupt growth factor signaling. We next wished to examine whether acidic pH would prevent the activation of these pathways during stimulation by serum.

**Figure 6:**
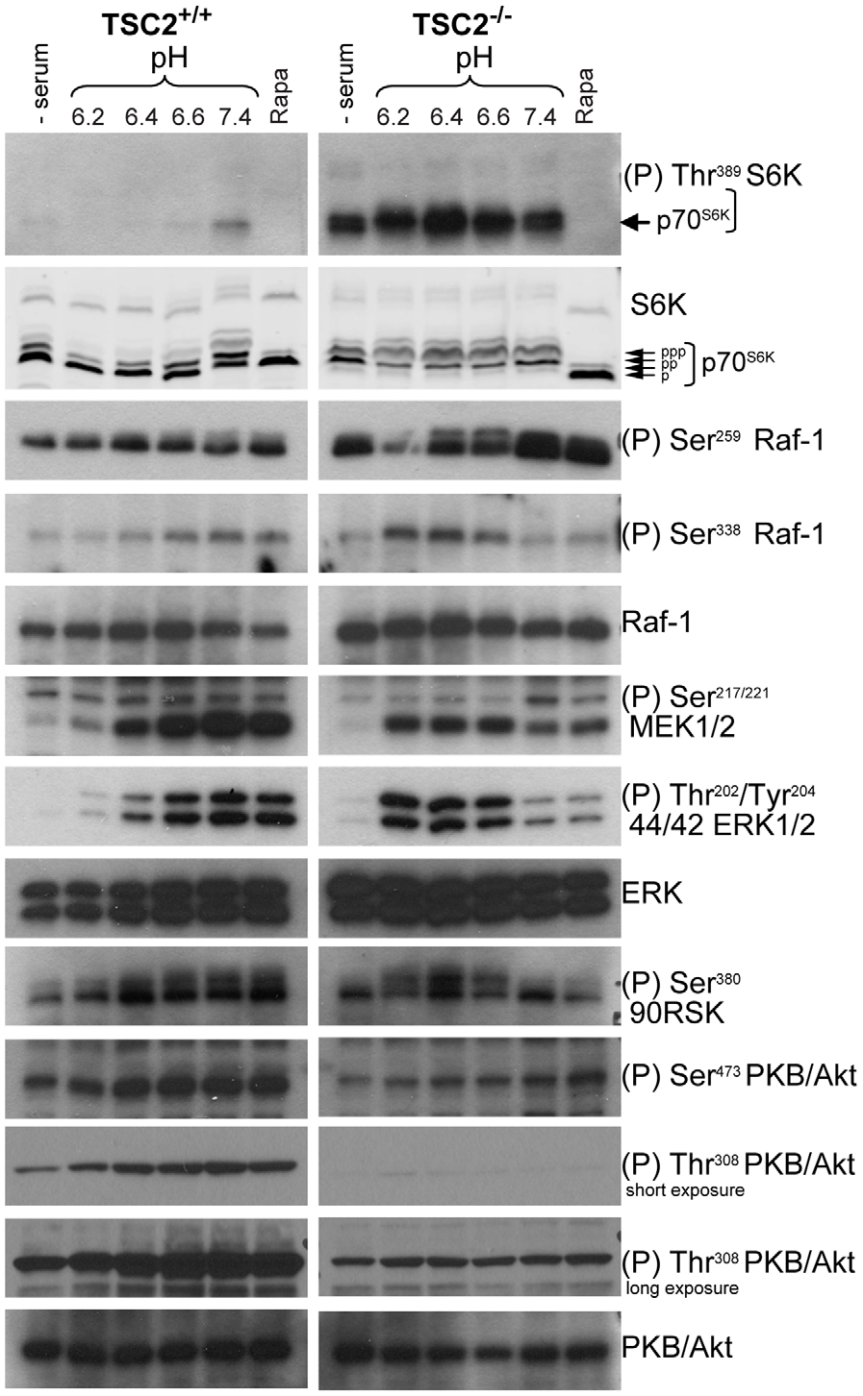
Effect of acidic extracellular pH on serum-induced activation of the mTORC1 pathway in TSC2^+/+^ and TSC2^−/−^ MEFs. Cells were incubated in serum-free medium for 18 h and then exposed for 1 h to serum-containing medium buffered to the indicated pH or to 30 nM rapamycin for 1 h. Lysates were analysed by western blotting using the indicated antibodies.

Serum starvation resulted in complete repression of mTORC1 activity in TSC2^+/+^ MEFs as measured by S6K phosphorylation, while mTORC1 was not inhibited by serum starvation in TSC2^−/−^ MEFs (Fig. 6), as previously reported [40], [41]. In the absence of serum, phosphorylation of ERK at Thr^202^/Tyr^204^ and MEK at Ser^217/221^ was essentially absent and phosphorylation of Raf-1 at Ser^338^ was reduced in both TSC2^+/+^ and TSC2^−/−^ cells (Fig. 6). In TSC2^+/+^ cells, addition of serum at pH 7.4 strongly activated Raf, MEK, ERK and mTORC1, as expected (Fig. 6). By contrast, serum showed a reduced ability to activate these kinases in acidic extracellular conditions (Fig. 6). In TSC2^−/−^ cells, addition of serum at pH 7.4 increased Raf, MEK and ERK phosphorylation but curiously, addition of serum at acidic pH caused a transient increase in phosphorylation of these three kinases that was larger than at physiological pH (Fig. 6).

**Figure 7:**
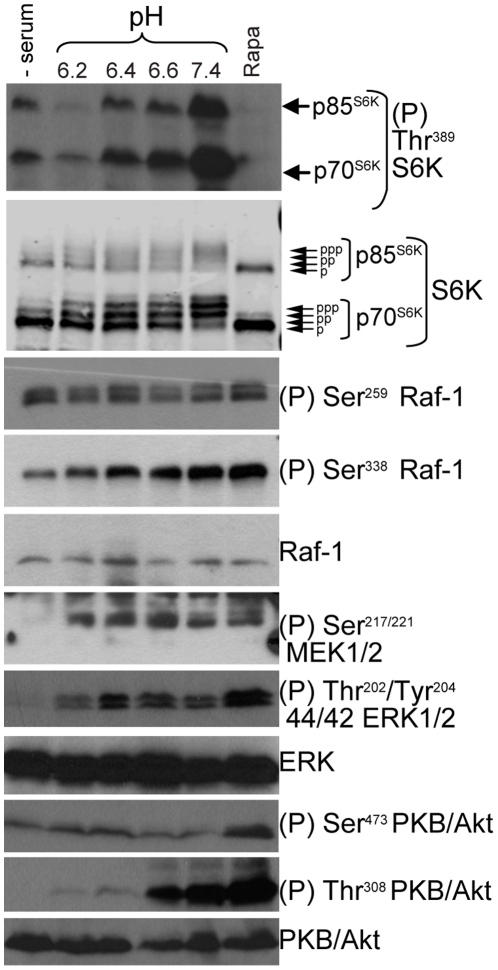
Effect of acidic extracellular pH on serum-induced activation of the mTORC1 pathway in MCF-7 cells. Cells were incubated in serum-free medium for 18 h and then exposed for 1 h to serum-containing medium buffered to the indicated pH or to 30 nM rapamycin for 1 h. Lysates were analysed by western blotting using the indicated antibodies.

In MCF-7 cells, serum withdrawal inhibited mTORC1 incompletely and serum was less able to stimulate mTORC1 at acidic pH than at physiological pH (Fig. 7). Phosphorylation of Raf, MEK and ERK was also considerably reduced by serum starvation and addition of serum was also less efficient at stimulating these kinases at acidic pH than physiological pH. Therefore, acidic external pH both inhibits active mTORC1 and curtails its activation by serum in the two cell lines.

### Involvement of TSC2 in downregulation of protein synthesis in response to acidification

**Figure 8:**
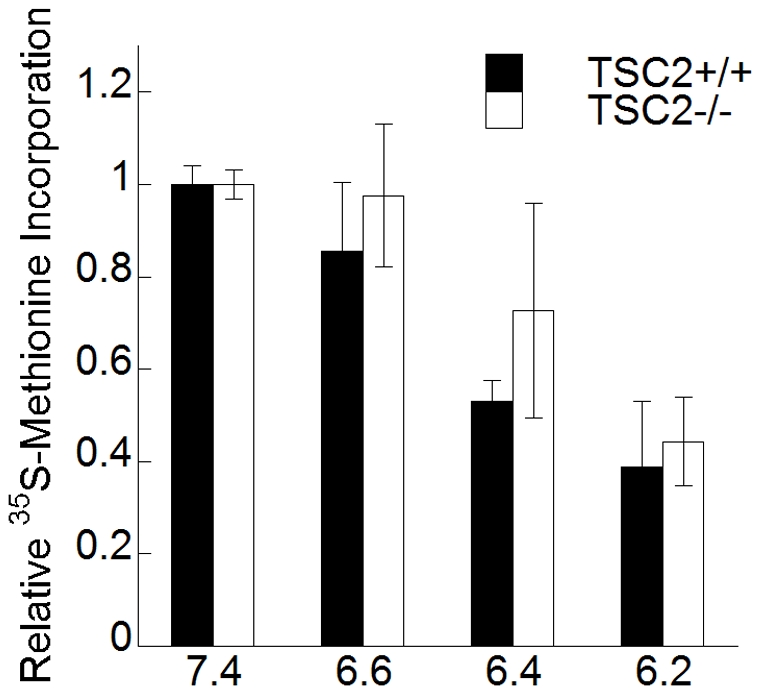
Effect of extracellular acidification on protein synthesis. The incorporation of ^35^S-Met into protein was measured in TSC2^+/+^ and TSC2^−/−^ MEFs exposed to cell culture medium buffered to the indicated pH values. The rates of ^35^S-Met incorporation were measured in the last 15 minutes of treatment to give cpm/µg of protein/15-min incubation. Results are expressed relative to the average incorporation in pH 7.4 medium. (mean ± S.D., n = 6).

Cellular acidification can negatively impact protein synthesis [42]. To investigate the dependence of this effect on the TSC1–TSC2 complex, TSC2^+/+^ and TSC2^−/−^ MEFs were exposed to complete medium at pH 6.2, 6.4, 6.6 or 7.4 for 1 h and protein synthesis was measured during the last 15 min of incubation. Protein synthesis was strongly reduced at pH 6.2 in both cell lines (Fig. 8). At pH 6.4 and 6.6, protein synthesis was only slightly more inhibited in TSC2^+/+^ MEFs than in TSC2^−/−^ MEFs.

## Discussion

mTORC1 signaling is regulated by a number of extracellular and intracellular cues including growth factors, nutrients, energy and oxygen levels [43], [44], [45], [46]. The main finding of this study is that mTORC1 signaling is additionally controlled by acidic pH. mTORC1 activity is inhibited rapidly, reversibly and in a graded fashion by acidic extracellular pH within the range observed in a variety of physiological or pathological conditions.

To shed light on the mechanisms involved in sensing and relaying pH information to mTORC1, we examined the effect of acidic extracellular pH on intracellular pH and on the major signaling pathways known to control mTORC1 [43], [47]. Exposure of cells to acidic medium caused rapid acidification of the cytoplasm. The rapid time course of both mTORC1 inhibition and intracellular acidification is compatible with pH sensing taking place inside the cell rather than exclusively from the cell surface. Extracellular acidification failed to inhibit mTORC1 in cells lacking TSC2, showing that activity of the TSC1–TSC2 complex is required for mTORC1 inhibition and possibly implicating the TSC1–TSC2 complex as a component of a pH sensing and signal transduction pathway.

A number of mTORC1 regulatory pathways are known to signal via the TSC1–TSC2 complex [43] and were examined in this study. Extracellular acidification did not affect Akt phosphorylation at either Thr^308^ or Ser^473^ in MEFs or MCF-7 cells, suggesting that signaling through Pdk1 and mTORC2 is not affected by pH. AMPK, which activates the TSC1–TSC2 complex, was not activated by acidic pH, indicating that mTORC1 inhibition was also not a consequence of ATP depletion. The rapid time course of mTORC1 inhibition by acidic pH, which occurs within minutes, also argued against an involvement of REDD1, which operates via changes in gene expression, and consequently on a much longer time scale.

The Raf-MEK-ERK pathway was the only mTORC1 control pathway signaling via the TSC1–TSC2 complex that was strongly inhibited by acidic pH, particularly in TSC2^+/+^ MEFs. This pathway can inactivate the TSC1–TSC2 complex through direct phosphorylation of TSC2 by ERK and Rsk [26], [48] (Fig. S1). Acidic pH inhibited activating phosphorylations of ERK and MEK. In addition, acidification reduced the phosphorylation of C-Raf at Ser^338^in MEFs, which is required for maximal activity. The identity of the kinase responsible for phosphorylating Raf is uncertain but may be Raf itself [28], [49], raising the possibility that acidic pH affects Raf-1 dimerization or oligomerization. Phosphatidic acid plays an important role in activation of the ERK cascade. The recruitment of Raf-1 to the membrane and its activation require binding to phosphatidic acid [50], [51]. The charge of phosphatidic acid is affected by pH and phosphatidic acid has been proposed as an intracellular cellular pH sensor [52]. However, extracellular acidification did not appear to prevent the association of Raf with membranes, indicating that inhibition of Raf-MEK-ERK signaling is not likely to be caused by an inability of phosphatidic acid to bind Raf when cells are exposed to acidic extracellular conditions. Importantly, complete inhibition of MEK-ERK by drugs inhibited mTORC1 only modestly in TSC2^+/+^ MEFs and not at all in TSC2^−/−^ MEFs and MCF-7 cells, indicating that although Raf-MEK-ERK signaling is sensitive to acidic pH, it is not the most important pathway controlling mTORC1 activity by acidification. The identity of the primary pH sensor responsible for controlling mTORC1 remains to be identified.

Acidification of the cytoplasm, the extracellular space and the blood is strongly associated with low tissue perfusion, low glucose availability and high energy expenditure [53], [54], [55]. The observation that acidic pH controls mTORC1 by a mechanism requiring the TSC1–TSC2 complex has interesting implications for understanding the response and adaptation of cells to acidic pH. Sensing of acidic pH may enable cells to rapidly reduce mTORC1 activity to temporarily restrain energy-consuming anabolic processes in response to a variety of metabolically stressful conditions.

## Materials and Methods

### Chemicals and antibodies

General laboratory chemicals were purchased from Sigma-Aldrich, Fisher Scientific and BDH, Inc. Rapamycin and PD98059 were from Calbiochem, PD184352 from LC Laboratories, Hoechst 33342 from Invitrogen, and propidium iodide from Sigma-Aldrich. Antibodies were from the following vendors: Cell Signaling Technology, phospho Thr^389^ S6K (#9205), phospho Ser^473^ PKB/Akt (#9271), PKB/Akt (#9272), phospho Ser ^380^ p90RSK (#9341), phospho Ser^217/221^ MEK1/2 (#9121), phospho Thr^308^ PKB/Akt (#9275), phospho Ser^259^ Raf-1 (#9421), phospho-p44/42 Thr^202/204^ MAPK (Erk1/2) (#9106), AMPK (#2603), phospho Thr^172^ AMPK (#2535), mTOR (#2972); Santa Cruz Biotechnology, Inc., S6K C-18 (#230), phospho Erk (#9106), Raf-1 E-10 (sc-7267), β-tubulin H-235 (#9104), Erk; Millipore, phospho Ser^338^ Raf-1(05-538); Upstate, 4G10 phosphotyrosine (Tyr(P)); Developmental Studies Hybridoma Bank, LAMP1 (H4A3); Invitrogen, Alexa fluor 488 goat-α-rabbit (#A11008) and Alexa fluor 568 goat-α-mouse (#A11004).

### Cell culture

MCF-7 cells [56] were maintained in RPMI-1640 medium (GIBCO, #31800-022) supplemented with 0.2% sodium bicarbonate, 10% (v/v) fetal bovine serum and 100 units/ml penicillin/streptomycin at 37°C in a 5% (v/v) CO2 humidified incubator. TSC2−/−/p53−/− and TSC2+/+/p53−/− MEFs were a generous gift of Dr. David Kwiatkowski [40] and were maintained in high glucose Dulbecco's modified Eagle's medium (DMEM) supplemented with 10% (v/v) fetal bovine serum, 100 units/ml penicillin/streptomycin and 2 mM L-glutamine (Sigma-Aldrich).

### Treatment with acidic media

RPMI-1640 medium without sodium bicarbonate, containing 20 mM 3-(N-morpholino)propanesulfonic acid (MOPS), 10% (v/v) fetal bovine serum and 50 units/ml penicillin/streptomycin was pre-equilibrated for 30 min in a 5% (v/v) CO2 humidified incubator at 37°C and adjusted to different pH values using HCl or NaOH. All experiments were carried out with freshly prepared media. Cells were seeded in 6-well plates at 1 million cells/well (MCF-7), 400,000 cells/well (TSC2+/+) or 200,000 cells/well (TSC2+/+) in normal culture medium and cultured overnight. The next day, the medium was replaced with medium buffered to the indicated pH values that had been pre-equilibrated in a 5% (v/v) CO2 humidified incubator at 37°C and cells were incubated for 1 h. For starvation experiments, cells were rinsed with serum-free medium and incubated overnight (∼18 h) in serum-free medium. The starved cells were treated with cell media buffered to various pH values for 1 h as above.

### Cell lysis and western blotting

At the end of the experiment, the cells were rinsed with serum-free ice-cold medium adjusted to the appropriate pH and were immediately lysed by scraping in cold 20 mM Tris-HCl pH 7.5, 150 mM NaCl, 1 mM EDTA, 1 mM EGTA, 1% (v/v) Triton X100, 2.5 mM sodium pyrophosphate, 1 mM β-glycerophosphate supplemented with fresh 1 mM Na3VO4, 1 mM dithiothreitol and 1× complete protease inhibitor cocktail (Roche Molecular Biochemicals) on ice. Lysates were pre-cleared by centrifugation at 18,000 g for 15 min at 4oC. SDS PAGE and immunoblotting were carried out as described [56]. The samples were separated on a 10% acrylamide gel containing 0.33% methylene bisacrylamide except for S6K phospho and S6K which were analyzed as described [56].

### Protein synthesis

TSC2^+/+^ and TSC2^−/−^ MEFs in 24-well plates at 100,000 cells/well and 50,000 cells/well respectively in normal culture medium and cultured overnight. The next day, the medium was replaced with medium lacking methionine and supplemented with dialysed fetal bovine serum buffered to the indicated pH values that had been pre-equilibrated in a 5% (v/v) CO_2_ humidified incubator at 37°C. After 45 min incubation, 5 µCi ^35^S-Met (Perkin Elmer #NEG709A001MC) was added for an additional 15 min. ^35^S-Met incorporation was measured as described [57].

### Intracellular pH measurement

The cDNA encoding mCherry was obtained from AddGene (Cambridge, MA). HindIII and BamHI sites were introduced at the 5′ and 3′ ends respectively of the mCherry coding region of mCherry by PCR, and ligated into the corresponding sites of the pde4GFP-N1 vector in frame with de4GFP [25]. MCF-7 cells, grown on glass-bottom dishes (MatTek, Ashland, MA), were transfected with mCherry/de4GFP fusion plasmid. The pH-sensitive de4GFP and pH-insensitive mCherry fluorescence was captured using an Olympus Fluoview confocal microscope by excitation with a 488 nm laser or 543 nm laser respectively. To calibrate mCherry/de4GFP fluorescence ratio to pH, Chinese Hamster Ovary (CHO) cells were transfected with mCherry/de4GFP and imaged by confocal microscopy under pH-clamp conditions using the high-[K^+^]/nigericin technique [58], [59]. In the calibration experiments, the fluorescence intensity of de4GFP displayed a sigmoidal relationship to pH between pH 5.0 and pH 9.0, and pKa was calculated to be 7.45, similar to the previously reported value. Between pH 6.75 and 8.0, the relationship between pH and mCherry/de4GFP fluorescence ratio was linear [25]. MCF-7 cells expressing mCherry/de4GFP were exposed to serum-containing media buffered to pH 7.4, 6.6, 6.4, or 6.2 and intracellular pH was monitored with time by measuring the mCherry/de4GFP fluorescence ratio. Experimentally measured ratios for each cell were converted to pH using the linear relationship between fluorescence ratio and pH determined in the calibration experiments.

### Immunofluorescence microscopy

Cells were seeded on 7× detergent-treated coverslips in 12-well plates at 300,000 cells/well (MCF-7), 130,000 cells/well (TSC2 ^+/+^) or 60,000 cells/well (TSC2^−/−^) in normal culture medium and cultured overnight. The next day, the cells were incubated for 1 h in normal culture medium or medium buffered to pH 6.4 or pH 7.4 or for 2 h in HBSS (Hanks Balanced Salt Solution, #37150, StemCell Technologies). At the end of the incubation period the cells were fixed in 3% paraformaldehyde for 15 min, permeabilized with 0.3% triton X-100 in PBS for 15 min, and blocked in 3% BSA in PBS for 30 min. The cells were incubated with antibodies to mTOR and LAMP-1 for 1 h and washed 3 times with 3% BSA in PBS. The cells were then incubated with secondary antibodies for 45 min and further washed twice with 3% BSA in PBS. DNA was stained with Hoechst 33342 (500 ng/ml) for 2 min, the cells were washed 3 times with distilled water and the cover slips were mounted on glass slides using Celvol. The cells were imaged using confocal microscopy.

## Supporting Information

Figure S1 **Summary diagram of the major pathways controlling mTORC1 via the TSC1–TSC2 complex and of the kinase phosphorylation sites examined in this study.** Binding of growth factors to receptor tyrosine kinases (RTK) activates the AKT and Ras/Raf/MEK/ERK pathways that phosphorylate TSC2 at multiple sites and inhibit the formation of TSC1–TSC2 complexes, thus de-repressing mTORC1 via Rheb-GTP. Exposure to acidic extracellular pH inhibits mTORC1 (S6K Thr^389^phosphorylation), ERK Thr^202^/Tyr^204^ phosphorylation, MEK Ser^217/221^ phosphorylation and Raf Ser^338^ phosphorylation. Larger red asterisks indicate stronger phosphorylation inhibition.
(TIF)

## References

[1] Fitts RH (1994). Cellular mechanisms of muscle fatigue.. Physiol Rev.

[2] Kraut JA, Madias NE (2010). Metabolic acidosis: pathophysiology, diagnosis and management.. Nat Rev Nephrol.

[3] Yao H, Haddad GG (2004). Calcium and pH homeostasis in neurons during hypoxia and ischemia.. Cell Calcium.

[4] Johnson BA, Weil MH, Tang W, Noc M, McKee D (1995). Mechanisms of myocardial hypercarbic acidosis during cardiac arrest.. J Appl Physiol.

[5] LaManna JC, Griffith JK, Cordisco BR, Bell HE, Lin CW (1995). Rapid recovery of rat brain intracellular pH after cardiac arrest and resuscitation.. Brain Res.

[6] Zhang X, Lin Y, Gillies RJ (2010). Tumor pH and its measurement.. J Nucl Med.

[7] Becelli R, Renzi G, Morello R, Altieri F (2007). Intracellular and extracellular tumor pH measurement in a series of patients with oral cancer.. J Craniofac Surg.

[8] van Sluis R, Bhujwalla ZM, Raghunand N, Ballesteros P, Alvarez J (1999). In vivo imaging of extracellular pH using 1H MRSI.. Magn Reson Med.

[9] Vaupel P, Kallinowski F, Okunieff P (1989). Blood flow, oxygen and nutrient supply, and metabolic microenvironment of human tumors: a review.. Cancer Res.

[10] Karuri AR, Dobrowsky E, Tannock IF (1993). Selective cellular acidification and toxicity of weak organic acids in an acidic microenvironment.. Br J Cancer.

[11] Pouyssegur J, Franchi A, L'Allemain G, Paris S (1985). Cytoplasmic pH, a key determinant of growth factor-induced DNA synthesis in quiescent fibroblasts.. FEBS Lett.

[12] Chambard JC, Pouyssegur J (1986). Intracellular pH controls growth factor-induced ribosomal protein S6 phosphorylation and protein synthesis in the G0----G1 transition of fibroblasts.. Exp Cell Res.

[13] Bravo R, Macdonald-Bravo H (1986). Effect of pH on the induction of competence and progression to the S-phase in mouse fibroblasts.. FEBS Lett.

[14] Musgrove E, Seaman M, Hedley D (1987). Relationship between cytoplasmic pH and proliferation during exponential growth and cellular quiescence.. Exp Cell Res.

[15] Chiche J, Ilc K, Laferriere J, Trottier E, Dayan F (2009). Hypoxia-inducible carbonic anhydrase IX and XII promote tumor cell growth by counteracting acidosis through the regulation of the intracellular pH.. Cancer Res.

[16] Wakabayashi I, Marumo M, Graziani A, Poteser M, Groschner K (2006). TRPC4 expression determines sensitivity of the platelet-type capacitative Ca2+ entry channel to intracellular alkalosis.. Platelets.

[17] Rubinsztein DC, Gestwicki JE, Murphy LO, Klionsky DJ (2007). Potential therapeutic applications of autophagy.. Nat Rev Drug Discov.

[18] Hall MN (2008). mTOR-what does it do?. Transplant Proc.

[19] Inoki K, Corradetti MN, Guan KL (2005). Dysregulation of the TSC-mTOR pathway in human disease.. Nat Genet.

[20] Huang J, Manning BD (2008). The TSC1–TSC2 complex: a molecular switchboard controlling cell growth.. Biochem J.

[21] Levraut J, Grimaud D (2003). Treatment of metabolic acidosis.. Curr Opin Crit Care.

[22] Sarbassov DD, Guertin DA, Ali SM, Sabatini DM (2005). Phosphorylation and regulation of Akt/PKB by the rictor-mTOR complex.. Science.

[23] Hresko RC, Mueckler M (2005). mTOR.RICTOR is the Ser473 kinase for Akt/protein kinase B in 3T3-L1 adipocytes.. J Biol Chem.

[24] Garcia-Martinez JM, Alessi DR (2008). mTOR complex 2 (mTORC2) controls hydrophobic motif phosphorylation and activation of serum- and glucocorticoid-induced protein kinase 1 (SGK1).. Biochem J.

[25] Diering GH, Mills F, Bamji SX, Numata M (2011). Regulation of dendritic spine growth through activity-dependent recruitment of brain-enriched Na+/H+ exchanger NHE5.. Mol Biol Cell.

[26] Ma L, Chen Z, Erdjument-Bromage H, Tempst P, Pandolfi PP (2005). Phosphorylation and functional inactivation of TSC2 by Erk implications for tuberous sclerosis and cancer pathogenesis.. Cell.

[27] Carracedo A, Ma L, Teruya-Feldstein J, Rojo F, Salmena L (2008). Inhibition of mTORC1 leads to MAPK pathway activation through a PI3K-dependent feedback loop in human cancer.. J Clin Invest.

[28] Zang M, Gong J, Luo L, Zhou J, Xiang X (2008). Characterization of Ser338 phosphorylation for Raf-1 activation.. J Biol Chem.

[29] Wellbrock C, Karasarides M, Marais R (2004). The RAF proteins take centre stage.. Nat Rev Mol Cell Biol.

[30] Kerkhoff E, Rapp UR (2001). The Ras-Raf relationship: an unfinished puzzle.. Adv Enzyme Regul.

[31] Hardie DG, Scott JW, Pan DA, Hudson ER (2003). Management of cellular energy by the AMP-activated protein kinase system.. FEBS Lett.

[32] Inoki K, Zhu T, Guan KL (2003). TSC2 mediates cellular energy response to control cell growth and survival.. Cell.

[33] Carling D, Sanders MJ, Woods A (2008). The regulation of AMP-activated protein kinase by upstream kinases.. Int J Obes (Lond).

[34] Sofer A, Lei K, Johannessen CM, Ellisen LW (2005). Regulation of mTOR and cell growth in response to energy stress by REDD1.. Mol Cell Biol.

[35] Brugarolas J, Lei K, Hurley RL, Manning BD, Reiling JH (2004). Regulation of mTOR function in response to hypoxia by REDD1 and the TSC1/TSC2 tumor suppressor complex.. Genes Dev.

[36] DeYoung MP, Horak P, Sofer A, Sgroi D, Ellisen LW (2008). Hypoxia regulates TSC1/2-mTOR signaling and tumor suppression through REDD1-mediated 14-3-3 shuttling.. Genes Dev.

[37] Korolchuk VI, Saiki S, Lichtenberg M, Siddiqi FH, Roberts EA (2011). Lysosomal positioning coordinates cellular nutrient responses.. Nat Cell Biol.

[38] Sancak Y, Bar-Peled L, Zoncu R, Markhard AL, Nada S (2010). Ragulator-Rag complex targets mTORC1 to the lysosomal surface and is necessary for its activation by amino acids.. Cell.

[39] Sancak Y, Peterson TR, Shaul YD, Lindquist RA, Thoreen CC (2008). The Rag GTPases bind raptor and mediate amino acid signaling to mTORC1.. Science.

[40] Zhang H, Cicchetti G, Onda H, Koon HB, Asrican K (2003). Loss of Tsc1/Tsc2 activates mTOR and disrupts PI3K-Akt signaling through downregulation of PDGFR.. J Clin Invest.

[41] Gau CL, Kato-Stankiewicz J, Jiang C, Miyamoto S, Guo L (2005). Farnesyltransferase inhibitors reverse altered growth and distribution of actin filaments in Tsc-deficient cells via inhibition of both rapamycin-sensitive and -insensitive pathways.. Mol Cancer Ther.

[42] England BK, Chastain JL, Mitch WE (1991). Abnormalities in protein synthesis and degradation induced by extracellular pH in BC3H1 myocytes.. Am J Physiol.

[43] Zoncu R, Efeyan A, Sabatini DM (2011). mTOR: from growth signal integration to cancer, diabetes and ageing.. Nat Rev Mol Cell Biol.

[44] Corradetti MN, Guan KL (2006). Upstream of the mammalian target of rapamycin: do all roads pass through mTOR?. Oncogene.

[45] Reiling JH, Sabatini DM (2006). Stress and mTORture signaling.. Oncogene.

[46] Avruch J, Hara K, Lin Y, Liu M, Long X (2006). Insulin and amino-acid regulation of mTOR signaling and kinase activity through the Rheb GTPase.. Oncogene.

[47] Menon S, Manning BD (2008). Common corruption of the mTOR signaling network in human tumors.. Oncogene.

[48] Ma L, Teruya-Feldstein J, Bonner P, Bernardi R, Franz DN (2007). Identification of S664 TSC2 phosphorylation as a marker for extracellular signal-regulated kinase mediated mTOR activation in tuberous sclerosis and human cancer.. Cancer Res.

[49] Udell CM, Rajakulendran T, Sicheri F, Therrien M (2010). Mechanistic principles of RAF kinase signaling.. Cell Mol Life Sci.

[50] Kraft CA, Garrido JL, Fluharty E, Leiva-Vega L, Romero G (2008). Role of phosphatidic acid in the coupling of the ERK cascade.. J Biol Chem.

[51] Andresen BT, Rizzo MA, Shome K, Romero G (2002). The role of phosphatidic acid in the regulation of the Ras/MEK/Erk signaling cascade.. FEBS Lett.

[52] Young BP, Shin JJ, Orij R, Chao JT, Li SC (2010). Phosphatidic acid is a pH biosensor that links membrane biogenesis to metabolism.. Science.

[53] Smallbone K, Gatenby RA, Gillies RJ, Maini PK, Gavaghan DJ (2007). Metabolic changes during carcinogenesis: potential impact on invasiveness.. J Theor Biol.

[54] Fukumura D, Xu L, Chen Y, Gohongi T, Seed B (2001). Hypoxia and acidosis independently up-regulate vascular endothelial growth factor transcription in brain tumors in vivo.. Cancer Res.

[55] Fischer B, Muller B, Fisch P, Kreutz W (2000). An acidic microenvironment inhibits antitumoral non-major histocompatibility complex-restricted cytotoxicity: implications for cancer immunotherapy.. J Immunother.

[56] Balgi AD, Fonseca BD, Donohue E, Tsang TC, Lajoie P (2009). Screen for chemical modulators of autophagy reveals novel therapeutic inhibitors of mTORC1 signaling.. PLoS One.

[57] Robert F, Gao HQ, Donia M, Merrick WC, Hamann MT (2006). Chlorolissoclimides: new inhibitors of eukaryotic protein synthesis.. RNA.

[58] Chaillet JR, Boron WF (1985). Intracellular calibration of a pH-sensitive dye in isolated, perfused salamander proximal tubules.. J Gen Physiol.

[59] Baxter KA, Church J (1996). Characterization of acid extrusion mechanisms in cultured fetal rat hippocampal neurones.. J Physiol.

