# Supplementary figures and images for: Regulation of mTORC1 Signaling by pH
Source: PLoS One. 2011 Jun 29;6(6):e21549. doi: 10.1371/journal.pone.0021549 (PMC3126813; doi:10.1371/journal.pone.0021549)

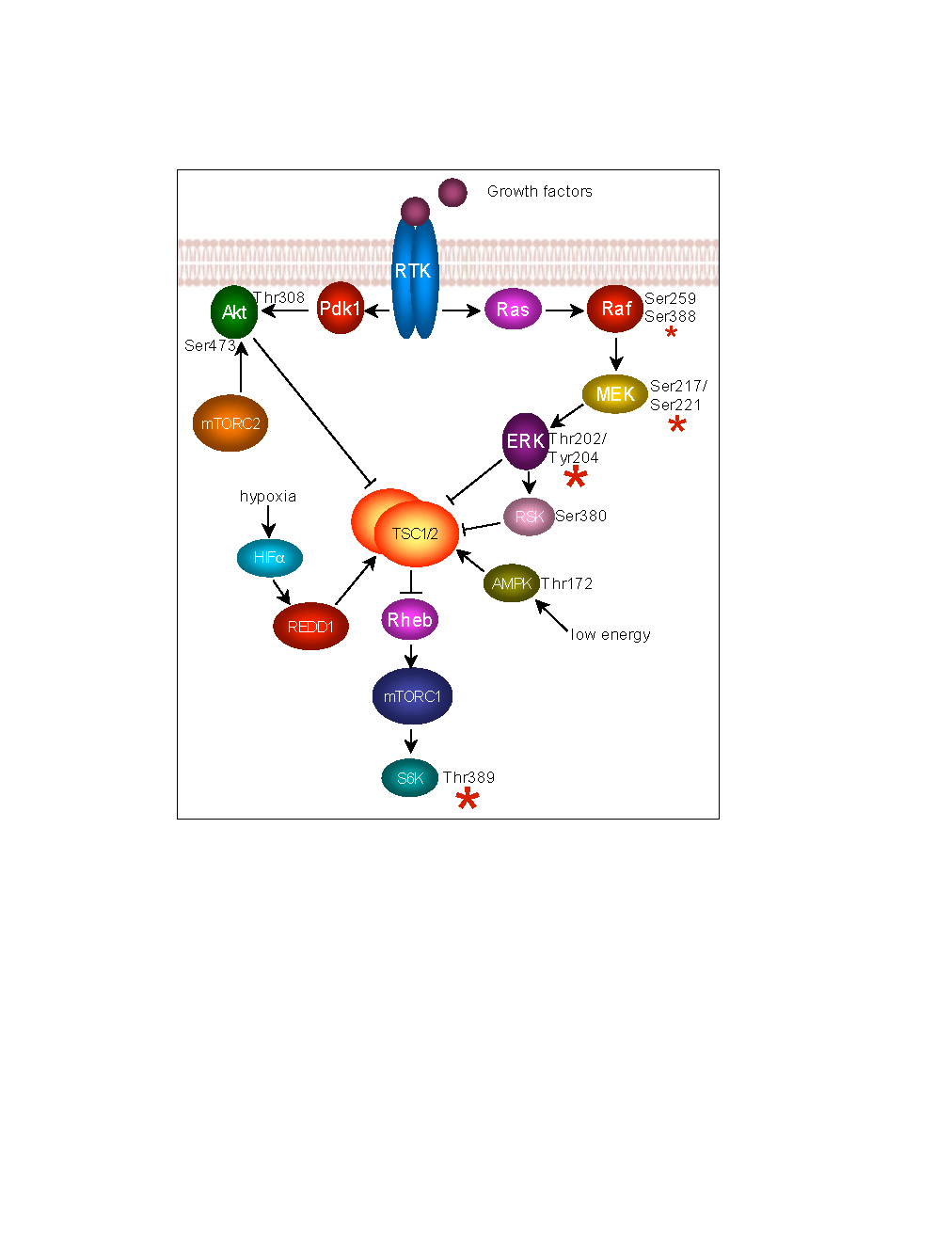

Supplement: Figure S1 — Summary diagram of the major pathways controlling mTORC1 via the TSC1–TSC2 complex and of the kinase phosphorylation sites examined in this study. Binding of growth factors to receptor tyrosine kinases (RTK) activates the AKT and Ras/Raf/MEK/ERK pathways that phosphorylate TSC2 at multiple sites and inhibit the formation of TSC1–TSC2 complexes, thus de-repressing mTORC1 via Rheb-GTP. Exposure to acidic extracellular pH inhibits mTORC1 (S6K Thr389phosphorylation), ERK Thr202/Tyr204 phosphorylation, MEK Ser217/221 phosphorylation and Raf Ser338 phosphorylation. Larger red asterisks indicate stronger phosphorylation inhibition. (TIF) [file pone.0021549.s001.tif]
